# Legal Cases in Lunacy

**Published:** 1853-04-01

**Authors:** S. Vallis Bone

**Affiliations:** Lincoln's Inn, Barrister-at-law


### LEGAL CASES IN LUNACY

AND IN CHANCERY, INVOLVING QUESTIONS OF INSANITY, ARGUED BEFORE THE LORD CHANCELLOR, THE LORDS JUSTICES OF THE COURT OF APPEAL, AND THE FULL COURT OF APPEAL IN CHANCERY.

*Reported exclusively for "The Journal of Psychological Medicine and Mental Pathology," by* S. Vallis Bone, Esq., *of Lincoln's Inn, Barrister-at-law.*

---

(Before the Full Court of Appeal in Chancery, the Lord Chancellor, and the Lords Justices Knight Bruce and Lord Cranworth, March 17 and 27, 1852.)

*In the matter of* Mrs. Catherine Cumming.

The title to traverse an inquisition by a person who has been found lunatic is a matter of right; but the Court, before granting the writ, will satisfy itself, by personal (or other) examination, that the alleged lunatic is able to exercise volition, and really desires to traverse the finding of the jury. In order to keep the expenses of a petition to traverse within proper limits, the Lord Chancellor ordered that, although eight were retained and were heard upon it, that the costs of two counsel only on each side should be allowed.

A commission *de lunatico inquirendo* had been issued against Mrs. Catherine Cumming, and was executed before Mr. Commissioner Barlow, the proceedings upon which we fully detailed at the end of Volume IV. of the *Psychological Journal*, when the jury returned a verdict finding her to be of unsound mind. Upon that, Mrs. Cumming presented a petition to the Lord Chancellor, praying leave to traverse the finding and for a stay of the proceedings in the lunacy in the meantime. The petition came on before the Lords Justices, but their Lordships, on the application of counsel, agreed that the case should be heard, if the Lord Chancellor consented, before the full Court, and Lord St. Leonards having signified his approbation, the same was so heard.

Mr. Bethell, Mr. Roundell Palmer, and Mr. Southgate supported the petition, contending that the traverse was a matter of right, and that all the discretion the Lord Chancellor had to exercise was to ascertain whether the alleged lunatic was capable of volition, and really wished to traverse the inquisition. The following cases and statutes were cited:—*ex parte* Roberts, 3 Atkins's Reports, 7; *ex parte* Barnsley, *ibid.*, 184; *ex parte* Southcote, 1 Ambler's Reports, 109; *in re* Fust, 1 Cox's Reports, 418; *ex parte* Ferne, 5 Vesey's Reports, 450, 832; Sherwood *v.* Sanderson, 19 *ibid.*, 280; and *ex parte* Ward, 6 *ibid.*, 579; *in re* Bridge, Craig, and Phillips's Reports, 338. The statutes:—17 Edward II., chap. 10; 8 Henry VI., chap. 16; 2 and 3 Edward VI., chap. 8; and 6 George IV., chap. 53.

The Attorney-General (Sir Frederick Thesiger), Sir W. P. Wood, Mr. Rolt, Mr. Petersdorff, and Mr. W. Morris, maintained the contrary view, insisting that there was no right at common law to traverse, and that in this Court the discretion of the Court could not be exercised, unless some ground were shown for supposing the finding of the jury to be erroneous. The following cases and statutes were relied on:—4 Coke's Reports, *folio*, 55, on Sir John Cutts' case; Ley's Reports, 26; Scowerfield's case, *ibid.*, 22; *ex parte* Duplessis, 2 Vesey's (senior) Reports, 555; Anonymous, Moseley's Reports, 71; Ludlam's case, 1 Collinson on Lunacy, 167; *ex parte* Saumarez, Secretary of Lunatics' Book, 1822, B. 44, No. 63, and Buller's *Nisi Prius*, page 216. The statutes:—34 Edward III., chap. 14; 36 Edward III., chap. 13; 1 Henry VIII., chap. 10; besides the statutes referred to on behalf of the petitioner.

Mr. Bethell was heard in reply.*

The Lord Chancellor (Lord St. Leonards).—The question which has been argued before us is one of great importance in a constitutional point of view, though it lies in a very narrow compass. There are two questions:—first, whether a party has a right to traverse the finding of a jury upon a commission against a person who has been found lunatic; and, next, whether the traverse is at all times "as of right." The true meaning of that expression I will consider afterwards. The whole question depends, in the first instance, upon the statute of Edward VI., and must be decided upon the words of that statute, fairly construed, and with regard also to the view of other Judges from the time of the passing of that ancient statute. Now, the sixth section of that statute, after providing for other cases, proceeds as follows:—"Or if any person be, or shall be, untruly founden lunatic, &c., be it enacted, that every person or persons grieved or to be grieved by any such office or inquisition shall and may have his or their traverse to the same, immediately or after, at his or their pleasure, and proceed to trial therein, and have like remedy and advantage, as in other cases of traverse upon untrue inquisitions or offices founden," &c. Much reliance has been placed upon these words, "and have like remedy and advantage, as in other cases of traverse upon untrue inquisitions or offices founden." Now, if in an application for a traverse, the Court is not simply at liberty, but is bound to enter into the whole question which was before the jury, it must hear all the evidence which was before the jury,

* It is impossible to give even an outline of the able and very learned arguments addressed to the Court in this very important case, but as many of the readers of the *Psychological Journal* may wish to refer to the authorities cited, they have been stated above. The judgments delivered by all the members of the Court are so plain and untechnical, that any abridgment of them would have been to diminish both the interest and value of the case, whether to the medical and legal, or the non-professional reader.

before it would be at liberty to grant or refuse the application. And in order to establish the claim to enter into the whole of the evidence, the case is put upon the ancient statute regarding escheators; and the proposition of the Attorney-General, founded upon that statute was, that there could be no right to traverse until you had established your title; and that being so as to escheats generally, he argued that the law, as applied to the present case, required, according to the words of the statute, that a party should have the like remedy and advantage as in other cases of traverse upon untrue inquisitions found; and, therefore, a title must be shown before the Court could grant an order to traverse. Now, that argument proceeds upon a mistake; for the right to traverse there given in regard to lands, and upon ordinary escheats and offices found, was of this nature: that the party had by the act not only a right to traverse, but to have the lands demised to him at a rent for a certain time during the existence of the trial. Now, this is explained very satisfactorily by Staunford, in his book upon "Prerogative." After stating several of these old statutes which have been referred to, he says:—"Hereupon it is to be noted that the showing of the evidence is only rehearsed as to the letting of the lands to farm, and not to the traverse; for by this statute he may traverse without showing any evidence, but not have the lands to farm. Also, by these statutes, he is not bound to any certain time for taking of his traverse, but only for taking of the lands to farm, for he may tend his traverse when he will, so he desire not the farm of the lands; but if he will have them to farm, he must tend his traverse within the month, as appeareth." So that in this book, which is always considered as one of great authority, it clearly appears that it is a misapplication of that statute to bring it to bear upon the statute which governs this case. Now, if this point had to be decided for the first time upon the meaning of the statute of Edward VI., I can hardly conceive that stronger words could have been used to give the party the right to traverse; it is to be "at his or their pleasure." It is, indeed, to be governed by the usual laws as regards traverses, as far as they may be applicable; but that does not take away the right given by these express words. The authorities of an early period do not much assist us. I will come down at once, therefore, to the time of Lord Hardwicke. In the cases referred to, though he did put it in some sense as a matter of right, yet it cannot be denied that he went into the cases and examined the circumstances to satisfy himself that it was a proper case for a traverse. How, then, did it come that the Chancellor was applied to? For, if it were an absolute right, the party might at once lodge his traverse in the Petty Bag Office. In a case which I shall presently state, that course was taken, and, though objected to, was not interfered with. I do not mean to say anything upon the question whether a party might not, behind the back of the Court, and irrespective of the statute 6 George IV., lodge his traverse at once in the Petty Bag Office. It is plain, however, how petitions came to be presented to the Court. The traverse cannot, of course, take place until the party has been found lunatic under a commission issued by the Great Seal. The Great Seal grants the custody of the persons and estates of lunatics. If a person goes behind the back of the Court and exercises his legal right to traverse (if he has such right), could he come and ask for his costs as upon a proceeding instituted under the authority of the Great Seal? He must depend upon his rights at law, whatever they may be. The reason, then, is evident why parties come with their petitions to this Court. The Chancellor has the duty of issuing this very writ, and therefore the parties come to ask permission to take this step; and, from all the cases, it appears that the Court has exercised, not so much a discretion, as a care and caution in issuing the writ. The authorities have all been referred to in the case of *in re* Bridge. Lord Hardwicke, I conceive, went to a greater length, in point of evidence, than any subsequent Judge would have gone. Lord Thurlow, *in re* Fust, following Lord Hardwicke, did go into the circumstances of the case, and I am not clear that he was wrong in so doing. In that case it became necessary to impeach a marriage. The husband came here to assist the traverse to prevent the marriage being impeached by the commission; and the Lord Chancellor, perceiving the collateral and improper purpose for which the traverse was to be used, refused to grant the order. As regards the latter cases, it is apparent that every successive Chancellor, from Lord Thurlow to the present time, has been of opinion that the granting of the writ is, in some sense, a matter of right. Lord Eldon's opinion as to the rule of law is perfectly clear; he states it again and again without limit; and, even in the case of a third party aggrieved by the finding, he is of opinion that he has a right to traverse. *Ex parte* Hall and in *ex parte* Ward, though the applicant was a stranger having no personal interest, he did not deny the right. The opinion of Lord Rosslyn upon the point admits of no doubt. Lord Lyndhurst was never called upon to express any opinion upon the point. Lord Brougham, in a case before him, *re* Tubb, in 1834, makes this observation:—"My predecessors seem to have considered it as matter of right." Lord Cottenham's authority was to the same effect. Lord Truro twice decided the point in accordance with the decisions of his predecessors. The authorities, therefore, as far as they go, are conclusive upon the question. In the case of Gervais Healy, which was in 1748, the party seems to have gone to the Petty Bag Office and entered a traverse without the intervention of the Court. The cousins and co-heirs of the lunatic petitioned the Court that the traverse might be discharged. It appears that no order was made, but the costs of the petition were reserved until the traverse should be tried. Before the traverse could be tried, Gervais Healy died. That seems to be a very considerable authority to show that, at that time, not only was it considered as of right, but so much so that the party might, behind the back of the Chancellor, file his traverse at the Petty Bag Office, and carry the same down for trial; but in that case the costs were to be reserved until the issue was tried. It appears from a case in a Lunacy Office in February, 1825, that a petition was presented by a lunatic for a traverse, and also a petition by the lunatic's son-in-law to be appointed committee; and thereupon an order was made by Lord Eldon, appointing an *interim* manager of the lands, and directing that the lunatic should be brought before him. A note was taken by Sir George Turner, V.C., of what fell from Lord Eldon upon that occasion. He said:—"I will see the person who is the subject of this commission, and learn from him whether it is his real intention to traverse." The effect of the decisions has been, that the party himself has a right to traverse, and also any other person having an interest in the question, but then he must do it within a limited time. And even with respect to the party himself, Lord Eldon says he is not so confident that there is not a right to limit him as to time. These authorities, therefore, are of the greatest weight, and it would require very great deliberation, and very strong reasons operating upon the mind of any Judge, to induce him to oppose his opinion to so much authority. But I am clearly of opinion that these decisions are authorized by the true meaning and spirit of the act itself upon which they are founded; and if I had had to decide the case now for the first time, I should have come to the same conclusion. Now, the traverse being as of right, the question remains how that right is to be exercised. It is impossible to deny that, in every case that has been cited, the Chancellor has addressed himself to the consideration of the question whether it was a proper case for the writ to issue; and if that was so, whether the determination of the question was made to depend upon an examination of the lunatic or the affidavits of parties. In order, then, satisfactorily to decide that question, you must first decide whether, in any proper sense, the writ is of right or not: because, if it is of right, as I think it will be now decided to be, then the considerations affecting the propriety of issuing the writ, or not, would be very different from those to which the Court would address itself if the writ were not strictly of right; but being strictly of right, it is still, as it ought to be, under a certain control. For instance, if, upon a petition for the writ, I were to call, as I necessarily must in every such case, for the attendance of the lunatic, and I saw instantly the signs of absolute raving madness, nobody could suppose that the person holding the office I fill would be at liberty to allow the writ to issue. In such case, it would be apparent that there was no untrue finding, and consequently no necessity for a traverse; and the duty imposed upon the Court of protecting the person and estate of the lunatic would require it to deny the writ. In some of the cases, before Lord Hardwicke particularly, and also before Lord Eldon, matters have been entered into in a manner hardly consonant with the strict interpretation of the term "as of right." But this is not matter of surprise, when one considers that a petition is presented and served, and the common order made that all parties should appear and be heard; but it is no proof of the right or duty of the Court to enter into the general question of the correctness of the finding of the jury. I cannot admit the authority of Lord Eldon for that proposition, when I find it deliberately stated, again and again, that it is of right, and that the Chancellor shall personally examine the lunatic upon the question. Now, this brings me to the consideration of my duty with reference to the decision in the present case. My first duty, without doubt, is to satisfy my mind that the application is *boná fide*, and that the lunatic is so far competent as to be able to satisfy me that she really does desire to have a traverse as against the finding, if I were in any doubt upon that point. I do not now mean to say that I would hear evidence as to her state of mind; but if I were not satisfied as to what her intentions and wishes were, if she herself did not appear to know her own mind upon the subject, I do not say that in such a case the Chancellor might not feel himself at liberty to consider by whom she was surrounded, by whom the application was made, and what were their objects and views. It will be time enough, however, to enter into these considerations when that question arises. The case now before me is one in which the Court will decide that the traverse is of right, in the proper sense of the term. I have myself examined Mrs. Cumming, and have represented to her what may be the consequences of this traverse in point of expense. I have had a considerable conversation with her, and, whatever may be her delusions upon other subjects, upon this point she is as reasonable, as free from heat or passion, as any reasonable person with whom I ever conversed. Though she is aware that it may put in peril the remainder of her property, she desires this traverse may issue. She is content to make this sacrifice, for, what she calls, her liberty of action; and she has satisfied me that, as far as such person can have volition, she has it. I am of opinion, therefore, that, the traverse being decided to be as of right, this is a case in which I am bound to allow it to go. I have one further observation to make, though it does not bear upon the question of law. The lady's property is said to be of small amount; without great caution the whole of it may be swallowed up in litigation, and, at the age of seventy-six, she may be stripped of the whole of her means of subsistence by the operation of a law brought in for her protection. It will be a reproach to all parties, and also to the law, if this matter is carried on with unnecessary expense. Upon this occasion, eight counsel have appeared; three on the part of Mrs. Cumming, and five on the other side. I make an order that the costs of two counsel only on each side be allowed; and I will make an order that, in whatever further steps are taken on either side, the expense shall be restricted within the smallest possible limit, in order to preserve to this lady, if it is in the power of the Great Seal to do it, some remnant of her fortune for the remainder of her days.

Lord Justice Knight Bruce: The observations of the Lord Chancellor render it almost superfluous for me to add anything. An inquest under a commission of lunacy is, I apprehend, in reality a proceeding to which the alleged lunatic is not a party, and therefore whether he shall have appeared before the jury for the purpose of confirming the allegation of lunacy or not, his rights after the finding of the jury are the same. If he is not in default for not appearing and contesting, then it would be a monstrous principle to hold that he is not entitled, as of right, to dispute the finding; nor do I understand law and principle, or, in other words, law and justice, to differ in that respect. The jurisdiction, therefore, in my judgment, is only to ascertain whether the application is an act of free-will on the part of the person, and not whether the will is that of a person of sound mind. When, in the judgment of that jurisdiction, there is a plain and sufficient expression of free-will, the traverse cannot, I think, be refused, however clearly beneficent to the alleged lunatic it may appear to the mind of the judge that the finding of the jury should remain undisturbed. It is the right of an English person to require that his personal freedom and the free use of his property should not be taken from him on the ground of alleged lunacy, without his being allowed the opportunity of establishing his sanity before a jury as a contesting party, and not merely as a subject of inquiry. Whether, in the present case, there has been such a plain and sufficient expression of free-will as has been mentioned (though I consider it highly probable), I do not know, and there I cannot give my opinion upon it, it having been thought right by the Lord Chancellor, Lord Cranworth, and myself, that the Lord Chancellor alone should see and converse with Mrs. Cumming.

Lord Justice Lord Cranworth: I have only to express my entire concurrence in the judgment of the Lord Chancellor and my learned brother, adding a few words to what has been said. I think that under the statute of Edward VI. the party aggrieved by an inquest which has found her a lunatic has a right to traverse; and that that right is clear under the words of the statute, independently of authorities. This right may appear inconsistent with the fact, that the Lord Chancellor exercises a sort of discretion. But that is a discretion to see that the party herself is really applying for the traverse; because the peculiar nature of the subject-matter of the inquiry makes the analogy from ordinary cases to a great extent inapplicable. If a person sues out a writ to recover land or other property, it is presumed to be his own act; but where there are *primâ facie* grounds for supposing the party to be incapable of having the will, it is fit that some precaution should be adopted for the purpose of seeing that that which is to be done as the exercise of the will of the party is really so done. This is ordinarily effected by the Lord Chancellor personally examining the party. I do not, however, mean to say, that even without such personal examination some inquiry might not be made on the subject; for example, where the person is in that state of bodily illness which would render it impossible that he should be seen by the Lord Chancellor. I do not say that a case might not occur in which some discretion might not be exercised; but in the general observations that have been made I entirely concur; and I have added these few words lest it should be supposed that I entertain some doubt, when I really entertain none.

(Before the Lords Justices of Appeal, April 2nd, 1852.)

*In the matter of* the same alleged Lunatic.

Committees will be appointed and a grant made notwithstanding a traverse, and the lunatic's income will be directed to be paid to her, as if the grant were not made. Arrangements for a lunatic's care and comfort will not be disturbed, although the Committees be appointed.

The full Court having decided that the traverse was of right, the remainder of Mrs. Cumming's petition, relating to other matters, came on now for hearing, together with a petition by her next of kin and co-heiresses at law, praying the confirmation of the Master's report, approving of certain persons as committees of the estate and the person. This Court, by orders dated severally in November and December 1851, has sanctioned arrangements for the personal care and comfort of Mrs. Cumming.

Sir W. P. Wood and Mr. Morris supported the second petition.

Mr. Bethell, Mr. Roundell Palmer, and Mr. Southgate for Mrs. Cumming.

Their Lordships saw no sufficient reason why the committees should not be appointed, nor why the grant should not be made; but directed that the income should be received by her as if the grant were not made; and ordered that the present system of personal care which had been sanctioned by order of the Court should not be interfered with until farther order.

---

(Before the Lords Justices of Appeal, July 17th, 1852.)

*In the matter of* Lord Dinorben.

Where a lunatic was tenant in tail in possession of family estates, and the Mastery in Lunacy had reported three persons as proper to be committees of the person and estate, the Court, on the petition of the tenant in tail in remainder, appointed him, in conjunction with the other three, to be committees of the estate. The mother of an infant next of kin of a lunatic, and as such next of kin entitled to a share of the savings of his property, is a proper person to be one of the committees of the estate.

In the matter of this lunacy there were two petitions, one presented by the present Lady Dinorben, the second wife of the late Lord Dinorben, as testamentary guardian and on behalf of her infant daughter by his lordship, praying the confirmation of the Master's report approving of her ladyship, Sir R. B. W. Bulkeley, and Mr. T. Peeres Williams as committees of the person and estates of the lunatic, the present Lord Dinorben, the late lord's only son by his former marriage. The other petition was presented by Mr. Hugh Robert Hughes, and nephew of the late and cousin of the present lord, praying that the report might not be confirmed, and that he might be appointed committee, or one of the committees, of the estate. From the affidavits it appeared, that Kinonel Park and other large estates were settled by the late Mr. Hughes upon the late father of the lunatic, Lord Dinorben, for life, with remainder to his first and other sons in tail, with remainder to Mr. Hugh Robert Hughes (since deceased) for life, with remainder to his first and other sons in tail. Under this settlement the lunatic was tenant in tail in possession, and Mr. Hughes, the petitioner, was tenant in tail in remainder. The two petitions came on together.

Sir W. P. Wood and Mr. Selwyn in support of the petition of Mr. Hughes contended, that the practice of the Court was that the remainder-man should be a committee of the estate; and relied upon *ex parte* Webb and Phillips's Reports, page 532, where Lord Cottenham laid down the principle that a remainder-man is entitled in the same way as an heir-at-law, to see that a lunatic's estate is well arranged. His lordship observed, "The primary object of the Court in these cases is to see that the lunatic's estate is well taken care of, and it cannot be denied that the remainder-man has a strong interest in its good management." But the argument here is, that by that very interest he is disqualified; and a distinction has been attempted to be drawn between a remainder-man and an heir-at-law. The distinction, however, if it be one, is merely technical; there is evidently no substance in it, at least in such a case as this, where the lunatic is sixty years old, and his recovery is all but hopeless. In many cases the object of the Court, to take care of the estate, could not be accomplished if those most interested in it were to be excluded upon considerations of that kind.

Mr. Bethell and Mr. Shapter argued, that the petition of Mr. Hughes was unnecessary, and that the report of the Master ought to be confirmed. They read from the affidavits a document in the hand-writing of the late lord, in which he stated his earnest desire, if the Lord Chancellor should consider a commission of lunacy necessary regarding his son, that Lady Dinorben and the two gentlemen who had been approved of as committees should be intrusted with that office. They contended, that a tenant in remainder under limitations in a settlement was not in the same or an analogous position to an heir of a lunatic.

Mr. Lloyd and Mr. Hislop Clarke appeared for the infant daughter, and followed in the same line of argument.

Sir W. P. Wood was heard in reply.

Lord Justice Lord Cranworth: The only doubt entertained by my learned brother and myself is whether Mr. Hughes should be associated with the three who have been approved of by the Master, or whether he should be one of the committees instead of one of those three individuals. Anciently the notion was, that the heir of a lunatic was an improper person to be intrusted with his person, because he might be supposed to be interested in getting him out of the way, as it was called, but that he was a proper person to be intrusted with the care of his estate, because he was most likely to manage it properly. Whatever may now be thought of the former part of the rule, there is good sense in the latter point; and the general practice of this Court is, that the heir, or *quasi* heir, or remainder-man, shall be the committee, or one of the committees; and for that reason, in large estates the Court confides the care to more than one person, and the question is, whether there is anything alleged or proved in the case now before us to take the case of Mr. Hughes out of the general rule? Undoubtedly there is not. As Lady Dinorben will, in all human probability, have the care of the lunatic, it seems right that the remainder-man should have some control over the care of the estate; and considering that the young lady, as next of kin, will be entitled to a share of any savings, I think that her mother, Lady Dinorben, should also have some control over the management of the estate. I am, therefore, of opinion that, under all the circumstances of the case, Mr. Hughes ought to be appointed one of the committees of the estate, with the three approved by the Master, while those three should be appointed as the committees of the person.

Lord Justice Knight Bruce: Believing, as I do, and satisfied, as I am, that Lady Dinorben is a lady unexceptionable in every point of view, still I do not hesitate to say, that my opinion would have been that Mr. Hughes, Mr. Williams, and Sir Richard Bulkeley should be appointed the committees of the estate, to the exclusion of her ladyship; but this is a point in which I think I may defer to the opinion of my learned brother, and say, that the committeeship of the estate should be intrusted to the four, while that of the person should be left with the three of whom the Master has already approved. I agree to this the more readily on account of the manner in which Lady Dinorben is mentioned in the writing left by her late husband. I very willingly concede my opinion in this matter to the opinion of Lord Cranworth.*

* The lunatic is since dead.

(Before the LORDS JUSTICES OF APPEAL, Aug. 4, 1852.)

(*In the matter of* BRIGGS.)*

The Court will not sanction the carrying on of a business of the lunatic, but will send a reference to the Master to inquire whether it will be for the benefit of the lunatic that it should be carried on.

The lunatic in this case was possessed of very small means, and a party who now presented a petition, offered to pay out of his own pocket so much as would make up the income to 30*l*. a year. The petition also stated, that the lunatic was owner of a ship and part owner of another ship, both carrying coals between Newcastle and the port of London, and it was proposed and prayed, that the committee might continue to carry on the business for the advantage of the lunatic.

Mr. C. J. SIMPSON appeared in support of the petition.

LORD JUSTICE LORD CRANWORTH: The offer of making up the income to 30*l*. a year seems to be a bribe to us, to sanction the committee in carrying on the shipping business of the lunatic. If we do that, why may we not in every case sanction the carrying on the business of a lunatic by his committee?

LORD JUSTICE KNIGHT BRUCE.—Of course we can do no such thing as is asked. Suppose the lunatic's ship to run down another, and suppose the Judge of the Admiralty Court to hold that the commander of the lunatic's ship is in the wrong, who is to pay in such a case? All that we can do is to refer the whole matter to the Master in Lunacy, to inquire and state whether the arrangement proposed or any part of it will be for the benefit of the lunatic.

---

(Before the LORDS JUSTICES OF APPEAL, Nov. 17, 1852.)

*In the matter of* RUTTER.

Where a lunatic's estate consisted of weekly rents a committee was permitted to receive them before perfecting his securities. The Court sent a reference to the Master to inquire whether it would be for the lunatic's benefit, that the business of his shop should be carried on.†

The Master in Lunacy had approved of a person as the committee of the lunatic: that an allowance of 20*l*. a year should be paid to his wife, and that 100*l*. a year should be applied for the maintenance of the lunatic himself. It appeared upon the evidence, that the commission had only issued in June, 1852, but that the lunatic had for seven years allowed his wife the above sum for her maintenance, they living apart. It also appeared that the lunatic was the owner of a chandler's shop, and it was alleged that to carry it on would be greatly for his benefit, and therefore it was asked that the Court would sanction the arrangement.

Mr. G. W. COLLINS appeared in support of the petition.

Mr. ROGERS appeared for the proposed committee, and stated, that it would be impossible to complete his securities in less than six weeks, and submitted that it would be of great benefit to the lunatic, if the committee were allowed to receive the rents, arising as they did from weekly tenancies, at once. The evidence in favour of the fitness of the committee was strong.

LORD JUSTICE KNIGHT BRUCE.—We think the report should be confirmed; and the committee undertaking to perfect his securities within six weeks from this time, let him be at liberty to receive the rents and pay 20*l*. a year from the 25th of March, 1852, to the wife, until further order, and let it be referred to the Master to consider and report on the propriety of carrying on the lunatic's business.

* See also the next case. † As to this, see last case.

(Before the Lord Chancellor, March 20th, 1852.)

*In the matter of* Burridge.

The Court refused to sell a reversion belonging to a pauper lunatic to pay the expenses of the commission. The object of a commission ought to be to protect the lunatic, and to provide for his sustenance and comfort.

The lunatic in this case was possessed of an income of 4*l.* a year, arising from the rents of a freehold cottage, and he was entitled to the capital of a reversion producing 256*l.* a year, the tenant for life of which was still alive. The father of the lunatic sued out the commission, and in doing so incurred costs to the amount of nearly 400*l.*, and another relation had advanced monies on account of his maintenance. The father subsequently became bankrupt, and the lunatic was sent to the workhouse, where an allowance of 2*s.* 3*d.* a day was made for his maintenance. An application was made by the father to the Master in Lunacy, for his sanction to a sale of the reversion, for the payment of all costs of the commission, and of the monies advanced for his benefit; but the Master refused to comply, whereupon the same person presented a petition to the Court for the same purpose.

Mr. Batten appeared in support of the petition.

The Lord Chancellor (Lord St. Leonards).—I refuse the prayer of the petition, as I will all others, where the interest of the lunatic has not been studied. The costs of this commission have been incurred very recklessly, and I am very dissatisfied with the mode in which the commission has been prosecuted. It is quite manifest that the proceedings have not been instituted with any view to the benefit of the unfortunate lunatic, but for the sole purpose of getting his property into the hands of other parties. The enormous sum of 400*l.* has been incurred in costs, at a time when it was well known that the whole of the property that the lunatic had in his possession was 4*l.* a year. The result is, he is allowed to go to the workhouse; and then the Court is asked to sell his reversion in order to pay these costs and a few other trifling sums for maintenance. The only doubt whatever that I entertain is in respect to these sums, which have been advanced by a relative for the maintenance of the lunatic; but, notwithstanding that it is proper that they should be repaid, the Court will not be justified in ordering the sale of the reversion on that account. As to the question of costs, there is nothing whatever to induce the Court to order a sale for their payment. The object of a commission ought to be to protect the lunatic, and provide for his sustenance and comfort; but have the present proceedings tended to such an object? The unfortunate lunatic has been sent to the workhouse on an allowance of 2*s.* 3*d.* a day, where he remains in an abject and degraded state, neglected by every one, while an application is made to sell his reversionary property for the payment of 400*l.* costs, and a few other debts, leaving a very poor chance of anything being saved from the wreck for his own benefit. I will not sanction any such proceedings, and the party petitioning, if he has any claim for costs, must wait until the reversion falls in, for the Court will not take any step for expediting their payment. The object of the commission was to get hold of this reversion, but I will take care that those views shall be defeated, and in the meantime will see whether something cannot be done for improving the condition of the lunatic, and at the same time to take care of his property. I will see that inquiries are made to that effect, and will ascertain whether arrangements cannot be made to remove the lunatic from the workhouse, and place him in a lunatic asylum, where he will receive proper care and nourishment; in the meantime I will make no order on the petition.

(Before the LORDS JUSTICES OF APPEAL, March 12 and 29, 1852.)

*In the matter of* Miss S. F. D. DAVIES, a person of unsound mind, and DAVIES *v.* DAVIES.

The Court directed part of certain funds in Court, to which a person of unsound mind, not found lunatic by inquisition, was entitled, to be invested in the purchase of a Government annuity, the income of the fund not being sufficient for her maintenance; the annuity to be bought in her name and for her life, and to be paid to her brother until the further order of the Court, he undertaking to apply the same towards her maintenance.

There was a suit of Davies *v.* Davies in the Court of Chancery, and Miss Sophia Frances D'Arley Davies was a defendant to it. She was a person of unsound mind, and had been so for upwards of seventeen years, although she was not found lunatic by inquisition. For that time she had resided at Brooke House, Clapton, under the care of Dr. Edward Thomas Monro. Her only property consisted of 600*l.* reduced annuities, to which she was entitled for life, and to one-seventh of it in reversion; from 762*l.* 10*s.* consols, and 41*l.* 19*s.* 6*d.* cash in the Bank, and a reversion in one-seventh of 3600*l.* consols, also in the Bank. The 762*l.* 10*s.* had only recently fallen in, and the dividends of the 600*l.* had alone been applied for her maintenance, her mother and brother and sisters having, during the whole time, made up the remainder out of their own monies. The mother had been appointed her guardian in the suit, and she, by her mother and by her brother, now petitioned the Court that the 762*l.* 10*s.* consols might be sold out, and the produce be applied in the purchase of a life annuity, under one of the National Debt Acts (48 George III., c. 142) in the joint names of the mother and brother, to be applied by them to the maintenance of the petitioner. The mother was a lady of seventy-nine years of age, and stated in her affidavit that she had no means of providing for her daughter.

Mr. STEVENS, in support of the petition. No opposition is offered to this arrangement, which must be for the benefit of the lady. It has been mentioned to one of the Vice-Chancellors who, having entertained some doubt upon a question of jurisdiction, has directed the petition to be set down here.

LORD JUSTICE KNIGHT BRUCE: As the petition is in a cause, the Vice-Chancellors have as much jurisdiction as we have; but it having been set down before us, we will, in order to save expense, hear it. We require, in the outset, to know whether anything so strong has been done as to deal with capital? The circumstances stated in the petition make the compliance with its prayer reasonable; and the only question is, whether the Court ought to deal with the principal money.

LORD JUSTICE LORD CRANWORTH: It would, in such a case, be a very wholesome jurisdiction to exercise. I, for my part, wish very much to do what is asked; but I do not recollect anything so strong being done. It is true it would only be carrying one step further what this Court is continually in the habit of doing with reference to applying part of the capital of an infant for payment of an apprenticeship fee when the fund is small. I think 600*l.* of this particular fund might be applied, leaving a small balance in case of necessity.

LORD JUSTICE KNIGHT BRUCE: Of course the name of a lady of seventy-nine cannot be used, nor would that of the brother be correct, who would most probably survive his mother, and he, moreover, appearing to be in trade. The annuity, at any rate, must be bought in the name of the lunatic, or perhaps it may be purchased in the name of the Accountant-General. The case can be mentioned again.

MARCH 29TH.

On this day, the Accountant-General having declined to permit the purchase in his name, an order was made for its purchase in the daughter's name by the brother for 600*l.* consols, part of the 762*l.* 10*s.*, from the Commissioners for the reduction of the National Debt, who were to pay the same to the brother until further order, he undertaking duly to apply it towards her maintenance.

---

(Before the LORD CHANCELLOR, March 20, 1852.)

*In the matter of* PROCTER.

Where the estate is small of which a lunatic is a trustee, and the Court has before it the same evidence as would be necessary before the Master, it will appoint a new trustee without a reference, in order to save expense.

Mr. KENYON PARKER appeared in support of a petition in this case, which prayed the appointment of a new trustee of a fund amounting only to 200*l.*, in the place of Mr. Procter, who was found lunatic. On account of the smallness of the amount it was asked that the appointment should be made without any reference to the Master. In answer to a question from the Court, the learned Counsel admitted that all the facts were not verified by affidavit, whereupon,

The LORD CHANCELLOR (LORD ST. LEONARDS) said: In this case I am asked to make an order, transferring property from one person to another, without a reference to the Master. This I would most willingly do to save expense, but then I must put myself in the place of the Master and examine the proofs. I cannot do so here, because the evidence is deficient, and in such cases I expect strict proof. The petition may be amended, but I shall not permit the estate to be charged for this day's attendance, and shall direct such costs to be disallowed.

---

(Before the LORD CHANCELLOR, March 20, 1852.)

*In the matter of* Mr. CHORLEY.

Before the Court parts with property vested in a lunatic, whether beneficially or as trustee, it will require the strictest proof of title.

Mr. Chorley, the lunatic, was sole trustee of a fund amounting to 800*l.* to which certain persons were absolutely entitled, among whom were married women. There was nothing to show that there had been no settlement or agreement for settlement on their marriage. A person, with a view to benefit the parties by preventing delay, paid the amount of the whole fund out of his own monies among the parties, who executed to him a release, and he now petitioned the Court for the transfer of the fund to him by way of repayment.

The LORD CHANCELLOR (LORD ST. LEONARDS), in ordering the petition to stand over, made the following observations: It behoves me, as guardian of the property of the lunatic, to exercise the greatest care before parting with it. Before making orders affecting the property of lunatics on *ex parte* applications, I shall require to be well satisfied that the interests of the lunatics will not be compromised by them; and, therefore, shall require, in such cases, the strictest evidence of title. In these cases I sit to exercise a most important jurisdiction, and that, too, in the absence of the parties whose rights are sought to be affected; and, in these cases, a grave responsibility rests with counsel as to the truth of the statements in the petitions.

---

(Before the Lords Justices of Appeal, March 30th, 1852.)

*In the matter of* Mr. Richards.

*Who may attend the execution of a Commission of Lunacy, and for what purpose.*

Where the object of a commission is to carry back the lunacy to a date anterior to the execution of a settlement, the parties interested in the settlement may attend the execution of the commission upon giving an undertaking to abide by any order of the Court as to costs, not only their own but the costs of the inquiry, so far as increased by such attendance.

Sir W. P. Wood and Mr. Locock Webb appeared in support of a petition presented by his next friend, praying for leave to attend the execution of an inquisition *de lunatico inquirendo* issued against a Mr. Richards, cousin of the infant, alleging a lunacy of thirty-five years. The object of the petitioner was to take care that the alleged lunatic was duly defended. Mr Richards, on the 14th of January, 1842, executed a deed of settlement of his property, by which, after reserving a life interest to himself, he gave a life interest to the petitioner's father, since deceased, then a life interest to the petitioner, and then he declared other trusts of the property. The commission was issued at the instance of a first cousin and of a third cousin of the alleged lunatic; and the petitioner was the child of a first cousin. The petition alleged that the petitioner had received no notice of the issue of the commission, but had only accidentally heard within the last three days that the same had issued; that an inquisition was to be held under the commission on the 1st of April; and that the petitioner feared that great injury would result to him, unless he were allowed to be represented at such commission.

The case *in re* Nesbitt, 2 Phillips's Reports, page 245, was cited, when Lord Cottenham said—" Such an application is in the discretion of the Court. It is for the interest of all parties that the truth should be ascertained, and, therefore, although it occasions a little additional expense, unless the estate be a very poor one, it is desirable that they (the petitioners who had an interest in the matter, somewhat akin to that of this infant) should have leave to attend."

Mr. Karslake: This petition, alleging as it does as a reason for its prayer that the petitioner fears, " that great injury will result to him if the execution of the commission of lunacy be not attended on his behalf," is bad on the face of it, and an order ought to be made. It is clear that the benefit of the lunatic is not intended, but the petition seeks the benefit of a third party. In the case of "*Ex parte* Snook, *in re* Watts," 1 Phil. 512, it was expressly held that the interest of the lunatic can alone be consulted in giving permission to attend the execution of the commission. The petition should be dismissed.

Lord Justice Knight Bruce: Speaking for myself, I should have been disposed, without the authority cited in support of the petition, to grant the prayer. My impression is, that as counsel I have been engaged to attend the execution of an inquisition for the sole purpose of seeing that the lunacy was not carried back beyond a certain date, my clients being no farther interested than in that fact. Here it is plain the purpose for which the attendance is sought. If the petitioners will undertake to abide by any order the Court may make as to costs, not only their own costs, but the costs so far as they may be increased by the attendance at the inquisition.

The undertaking was given and the order was made, as prayed.

(Before the LORDS JUSTICES OF APPEAL, April 30th, 1852.)

*In the matter of* MR. JOHN ROSE SWINDELL.

*Committee of a lunatic answerable for losses occasioned by his neglect.*

It is objectionable for the committee to employ as his solicitor in the lunacy a person who has lately been a bankrupt. It is also objectionable to let a part of the lunatic's estate to such solicitor. Rent having been in arrear, and the committee having neglected to distrain or to put in force a judgment given for the amount, and the money having ultimately been lost, the Court held the estate of the committee, who had died, answerable for the whole amount.

The Master in Lunacy having reported that a sum of money mentioned in the report, and with which the estate of the late committee was sought to be charged, had not been lost by the wilful neglect or default of the committee, and that therefore his administrator ought not to be charged with it, the case came before the Court for the confirmation of that report. The facts, although contained in a very voluminous report, may, so far as necessary, be shortly stated. Mr. John Rose Swindell was found lunatic in February, 1828, and Mr. Pearsall was appointed committee of the person and estate, and so continued until his death in November, 1838, Mr. Balguy acting as his solicitor in the matter of the lunacy the whole time. During this period—namely, in February, 1838, Mr. Balguy was declared bankrupt, but obtained his certificate in May following. After the death of Mr. Pearsall there ensued a strong contest for the committeeship, in which Mr. Balguy acted as solicitor for one of the competitors, who was ultimately successful, Mr. Edward Ordish, who was appointed in March, 1839. The costs of these proceedings were fixed at 98*l.*, and were paid by Mr. Ordish. From the time of his appointment till October, 1842, Mr. Ordish employed Mr. Balguy as his solicitor, both in the lunacy and in his private matters. Part of the lunatic's estate consisted of Borrowash House, which, being untenanted, Mr. Balguy offered to occupy as yearly tenant at 132*l.* a year, the rent paid by the last tenant, and to this offer Mr. Ordish acceded. Mr. Balguy transacted business for Mr. Ordish both in the lunacy and otherwise, and had claims for costs, and he paid no rent, and 395*l.* being due, Mr. Ordish distrained for the amount, whereupon Mr. Balguy executed a warrant of attorney for the amount and the distress was withdrawn. Mr. Balguy paid down 100*l.*, and subsequently 79*l.* was raised by a sale of some hay on the premises, and it was alleged that the reason for the withdrawal of the distress was that the goods were worth less than the 100*l.* Mr. Balguy paid down, and which would not have been paid excepting on that condition, and Mr. Balguy also agreed to pay the balance at 100*l.* a year. Fresh rent accrued before Mr. Balguy quitted the houses, and ultimately upwards of 300*l.* was lost, Mr. Ordish having abstained from enforcing the judgment on the warrant of attorney, it being stated in an affidavit filed on his behalf, that the Master had had that fact brought before him, and had acquiesced in such a course.

Mr. Ordish died in 1851, intestate, and Mr. J. P. Ordish, one of his sons, obtained letters of administration to his estate, and the committee having left property, the real question was, whether or not such property was liable to make good the loss of rent due to the estate of the lunatic?

Mr. SWANSTON and Mr. SMYTHE argued, that the report, having found that no wilful neglect or default had been committed, ought to be confirmed.

Mr. ROLL and Mr. J. V. PRIOR opposed the adoption of the report, and insisted that the administrator must make good all losses; but they were willing he should be allowed the 98*l.* costs Mr. Ordish had paid to Mr. Balguy on account of the lunacy.

Lord Justice Lord Cranworth: This is a very distressing, and a very objectionable case. This committee chooses to employ as his solicitor a gentleman who had been but a few months before a bankrupt, and had re-established himself. The first act done by the committee after that is, that he lets this house to his own solicitor—a very objectionable proceeding, at a rent of 132*l.* per annum. The solicitor continues tenant for four years, and never pays a sixpence of rent. Being the solicitor to the committee up to nearly the fourth year of his tenancy—or rather to the end of the third year —about the third year he is dismissed, and continues tenant for a year afterwards. The question is—whether, there having been no rent received, except a certain sum on account, that was not lost through the fault of the committee, in not enforcing its payment. The only excuse is, that these accruing costs, which is put into his mouth by the solicitor himself, putting that as a sort of set-off against the payment of rent. Eventually there are no costs, except 98*l.*, which the parties are willing to allow to be deducted, and the whole of this rent, except two sums of 100*l.* and 79*l.* is lost. No other conclusion can be arrived at than an answer in the affirmative to the question, I have stated, because the committee has been wanting in that vigilance which the Court is bound to require from one holding that fiduciary situation. I am extremely grieved that such is the result at which I am bound to arrive; but, considering how important it is to keep strict guard upon the conduct of persons undertaking to watch over those who by the visitation of God are incapable of taking care of themselves, there is no other conclusion to be arrived at but to charge the committee with the whole amount of 395*l.*, but to give him credit for the 100*l.* and the 79*l.*, then adding another year's rent, after deducting from it 98*l.* the amount of costs Mr. Ordish paid to Mr. Balguy.

Lord Justice Knight Bruce concurred.

---

(Before the Lord Chancellor. May 5th and 26th, 1852.)

*In the matter of* Miss Sophia Wheeler.

*A petition for vesting the estates held by a lunatic mortgagee in the mortgagor, on his paying off the money, ought to originate with the committee.*

Mr. Ebenezer Young mortgaged a leasehold property to Miss Sophia Wheeler, who afterwards became lunatic. A committee was appointed of her estate—Mr. Robert Lankester. Mr. Young being desirous of paying off the mortgage money, petitioned, under the Trustee Act, 1850, for leave to pay the money into Court, and for an order vesting the estate in him. On the 20th of March the order was made by the Lord Chancellor, but no order was made as to the costs. The committee was also a party to the petition.

Mr. Giffard, for the petitioners, asked that the costs of this matter might be paid, not by the mortgager, Mr. Young, but out of the lunatic's estate; and, as an authority, cited *in re* Lewes, in vol. 1 of Macnaghten and Gordon's Reports, page 23, where a mortgager was held liable to pay the costs, but that was because he knew at the time of the existence of the lunacy, which was quite different from the present case, for the lunacy occurred after the mortgage. In *ex parte* Richards, 1 Jacob and Walker's Reports, page 264, Lord Eldon held that, where a mortgager or committee of a lunatic presents a petition, the lunatic being beneficially interested in the mortgage money, the lunatic's estate must bear the costs of the proceeding, to enable the committee to re-convey. The learned counsel also cited *in re* Marrow, Craig and Phillips's Reports, page 142, and *in re* Townsend, 2 Phillips's Reports, page 348, and submitted that the situation of this lunatic was similar or analogous to that of a lunatic trustee.

Mr. Pitman appeared for the next of kin.

May 26th.

The Lord Chancellor (Lord St. Leonards). The case of *ex parte* Richards, before Lord Eldon, seems to dispose of this, and especially as the committee joins with the mortgagor in the petition. Although that case has been doubted (and certainly I originally should not have made such an order), yet as I found, both from reported and unreported cases, that it has been repeatedly followed, I do not feel myself at liberty to disturb it. I think it, however, contrary to principle, and opposed to the settled rule, that the costs occasioned by the mortgagee's putting the mortgaged estate into settlement, are to be paid by the mortgagor. I think, however, that the proceedings in such cases as this should originate with the committee, and not with the mortgagor; and in future, therefore, unless the committee declines to present a petition, I shall not give the mortgagor his costs.

